# Supplementary material for: Analysis of translating mitoribosome reveals functional characteristics of translation in mitochondria of fungi
Source: Nat Commun. 2020 Oct 14;11:5187. doi: 10.1038/s41467-020-18830-w (PMC7560712; doi:10.1038/s41467-020-18830-w)
Supplement: Supplementary file 1 — Supplementary Information [file 41467_2020_18830_MOESM1_ESM.pdf]

Supplementary Information

Analysis of translating mitoribosome reveals functional characteristics of protein synthesis in mitochondria of fungi

Yuzuru Itoh[1,2†], Andreas Naschberger[1†], Narges Mortezaei[1†], Johannes Herrmann[3] and Alexey Amunts[1,2]*

[1]Science for Life Laboratory, Department of Biochemistry and Biophysics, Stockholm University, 17165 Solna, Sweden

[2]Department of Medical Biochemistry and Biophysics, Karolinska Institutet, 17165 Solna, Sweden

[3]Cell Biology, University of Kaiserslautern, Erwin-Schrödinger-Straße 13, 67663 Kaiserslautern, Germany

† These authors contributed equally to this work.

*Correspondence and request for material should be addressed to amunts@scilifelab.se

This file includes:

Supplementary Fig. 1 to 18

Supplementary Tables 1 to 3

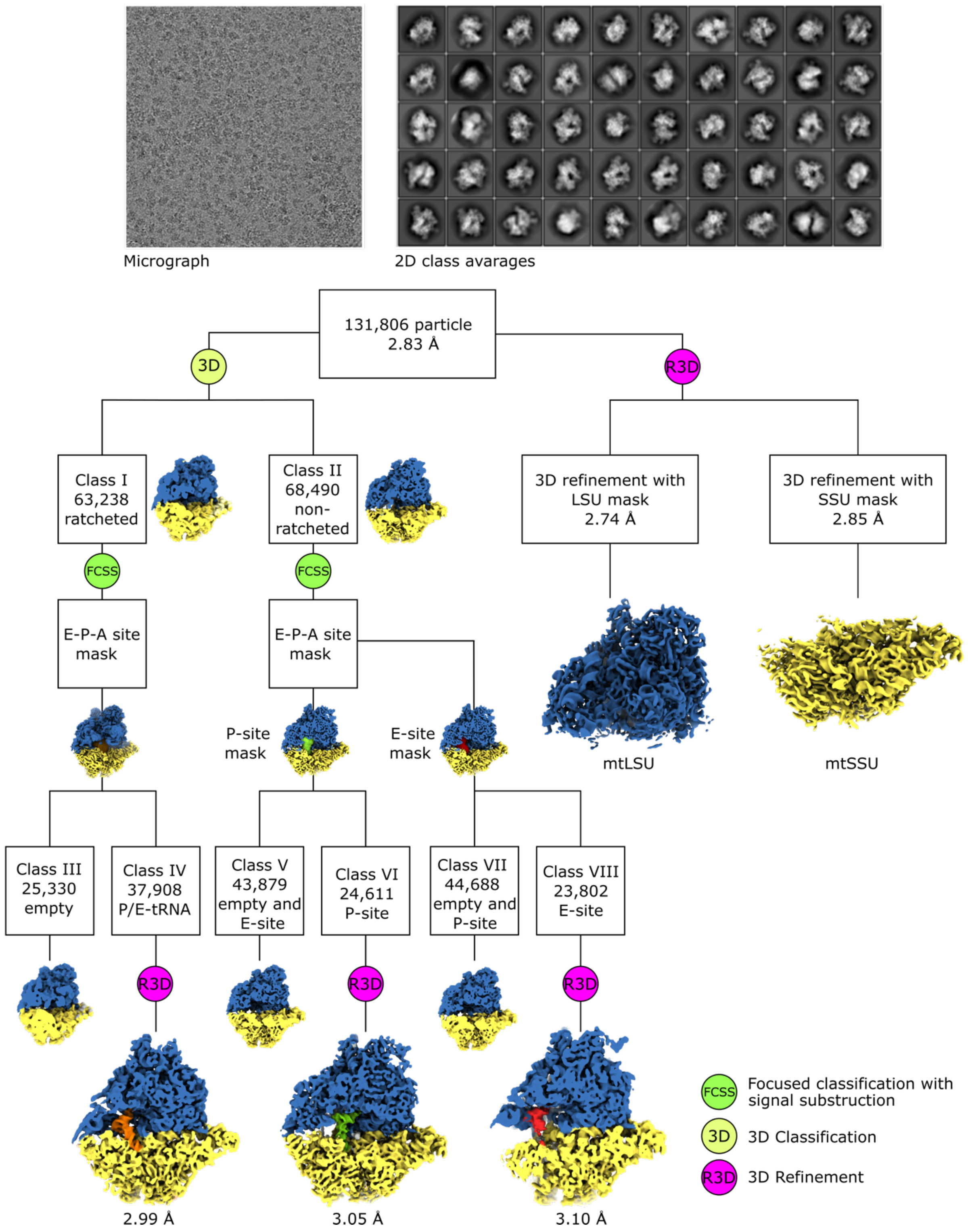

Supplementary Fig. 1: Cryo-EM data collection and processing scheme. Representative micrograph and 2D class averages. Data refinement protocol and classification scheme for mtLSU, mtSSU and monosome in three tRNA bound states.

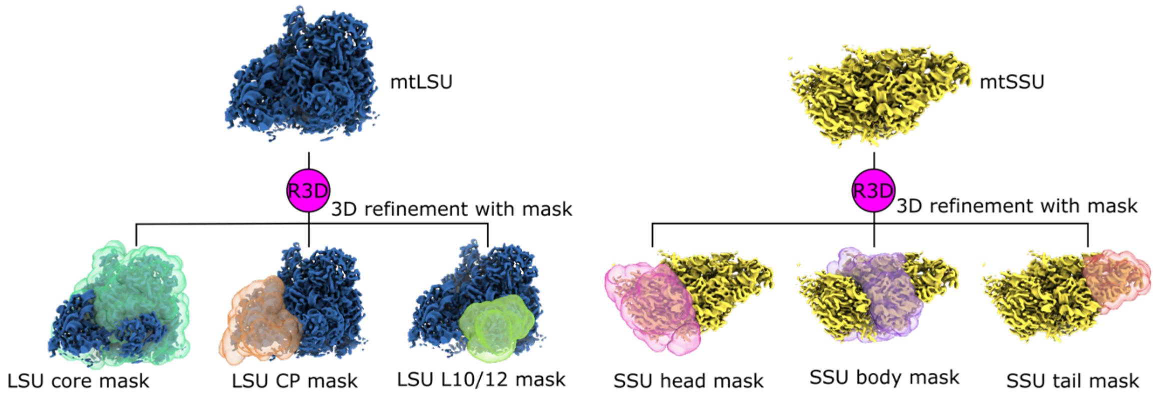

Supplementary Fig. 2: Masked refinement of mitoribosomal subunits. Masks for mtLSU core, CP, L7/12 stalk; mtSSU head, body, tail were used to improve the density locally.

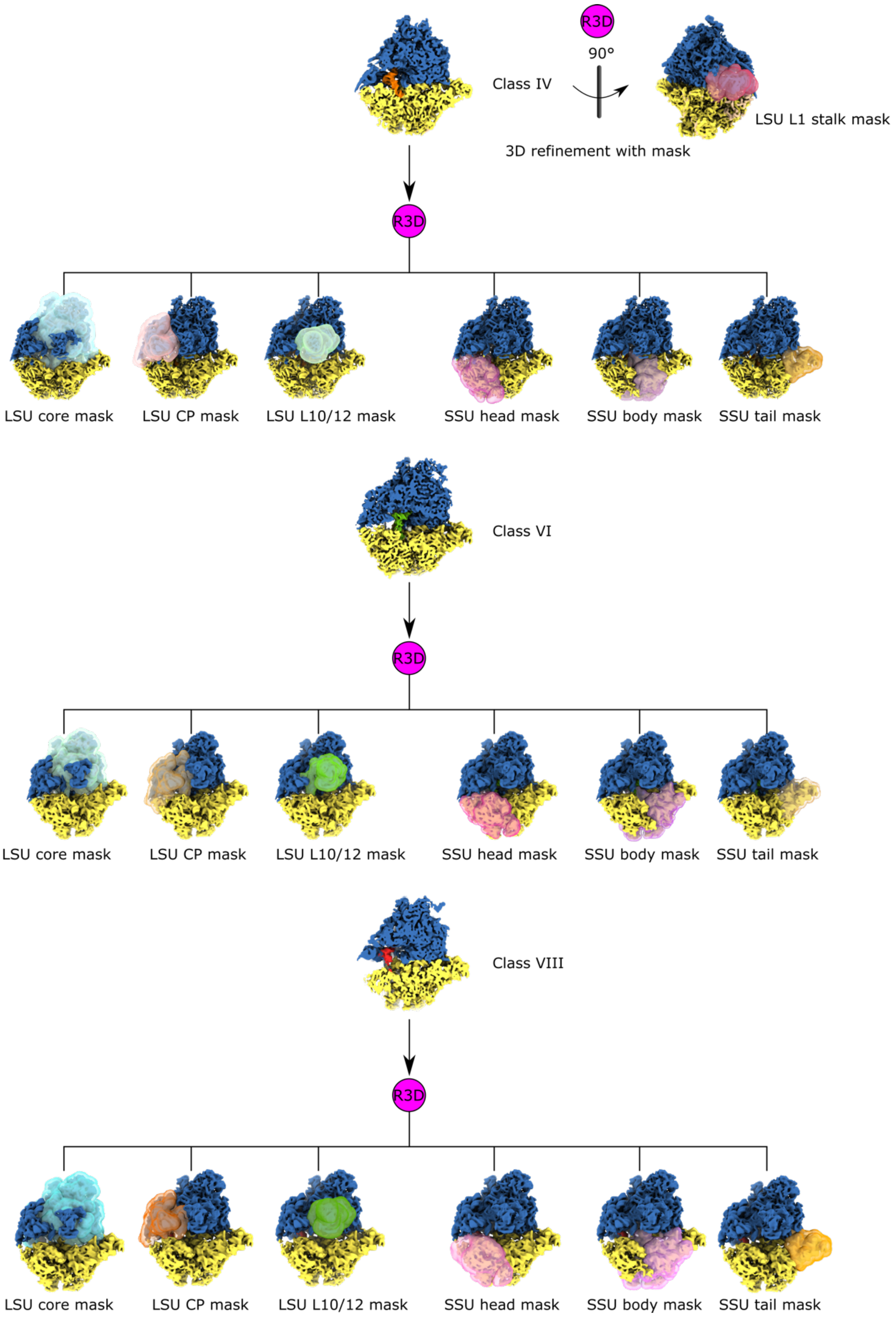

30

31 **Supplementary Fig. 3: Masking refinement of the mitoribosomes in tRNA bound states.**

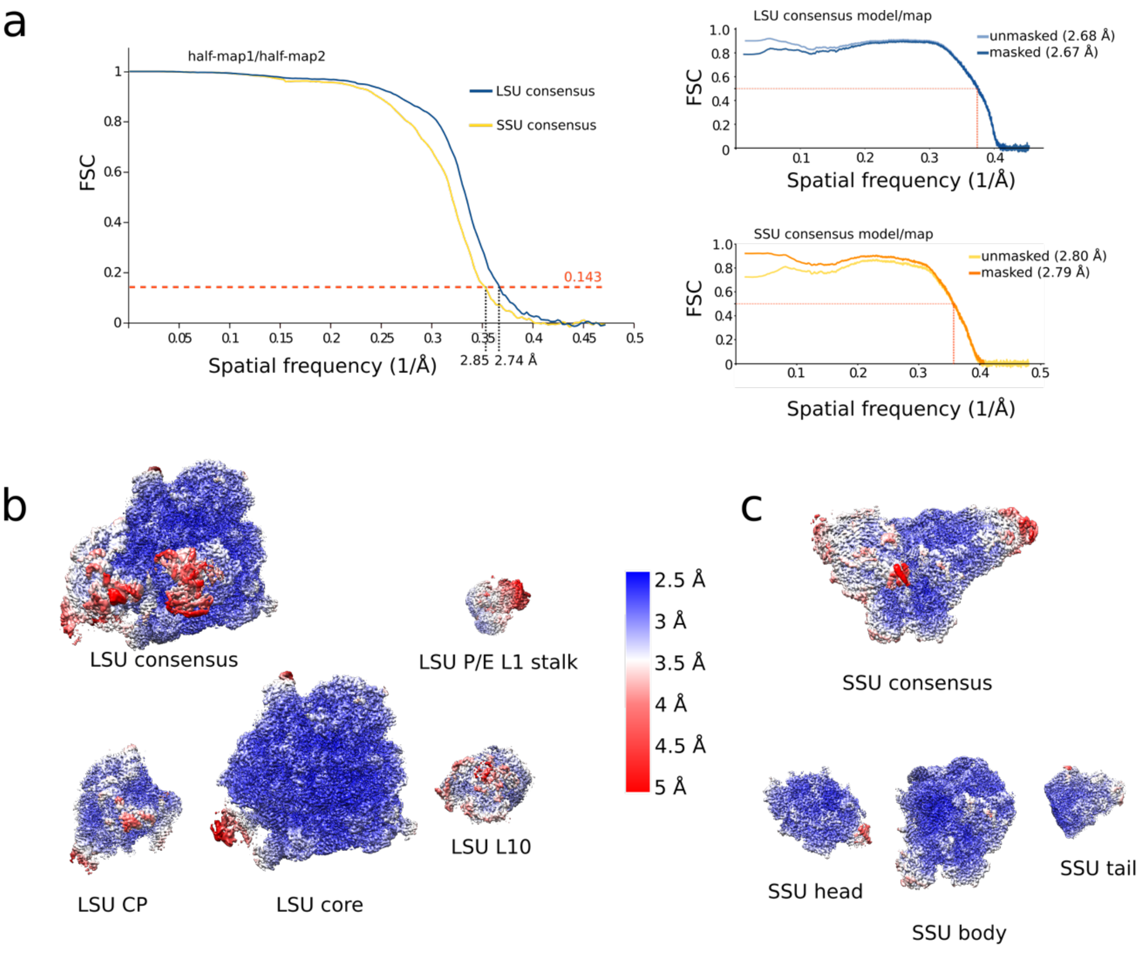

Supplementary Fig. 4: Overall and local resolution estimations of mitoribosomal subunits.a Fourier Shell Correlation (FSC) curves of half-maps and map-to-model are shown. Cut-off values of 0.143 (gold-standard) and 0.5 (model-to-map) are indicated and the corresponding resolution estimates are shown **b** mtLSU cryo-EM maps viewed according to local resolution. **c** mtSSU cryo-EM maps viewed according to local resolution.

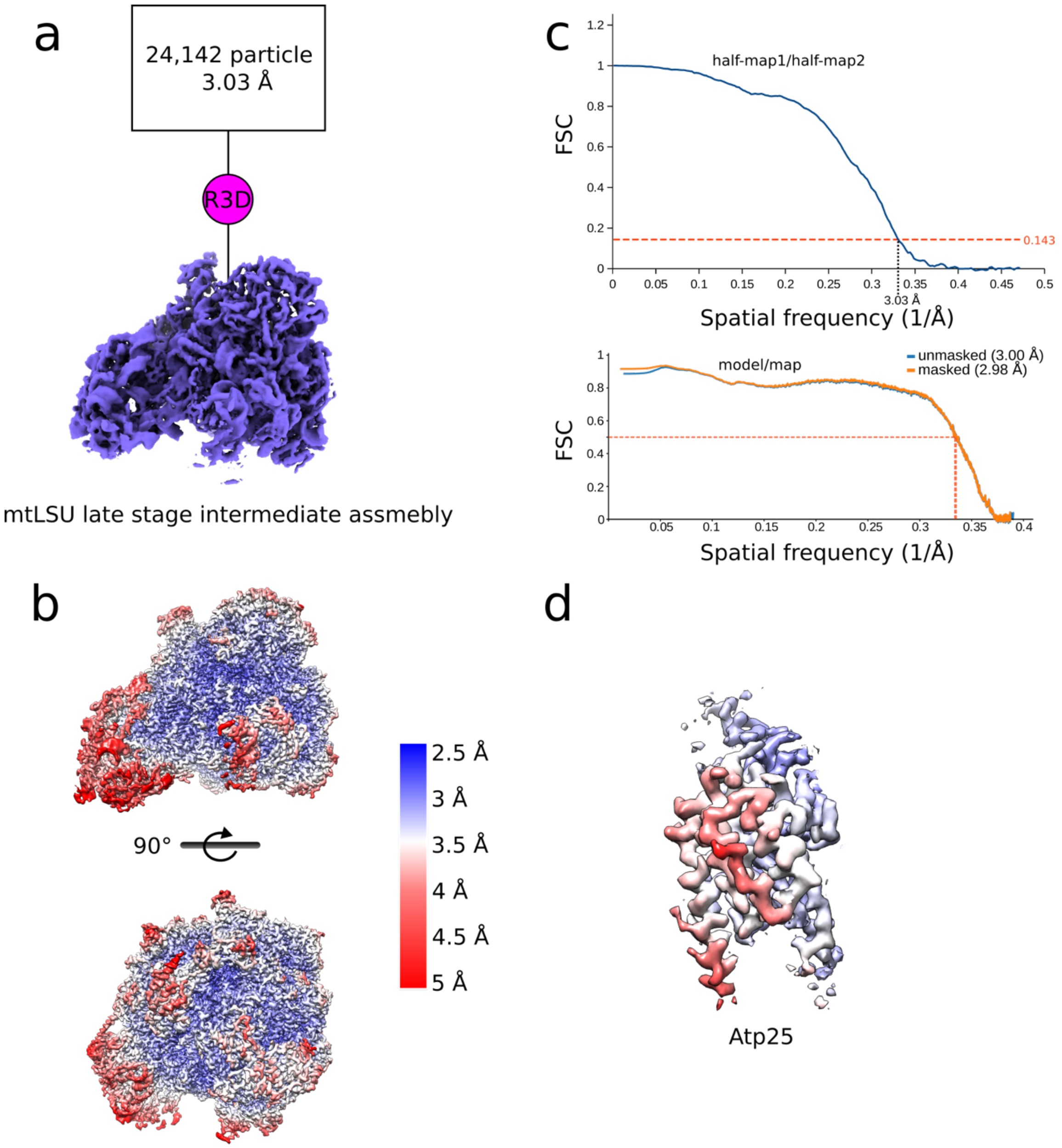

Supplementary Fig. 5: Data processing and overall and local resolution estimations of mtLSU-Atp25 assembly intermediate. **a** Processing scheme. **b** Fourier Shell Correlation (FSC) curves for half-maps and map-to-model are shown. Cut-off values are indicated and resolution estimates are shown. **c** mtLSU-Atp25 assembly intermediate cryo-EM maps colored according to local resolution. **d** Zoomed in map for Atp25 colored by local resolution.

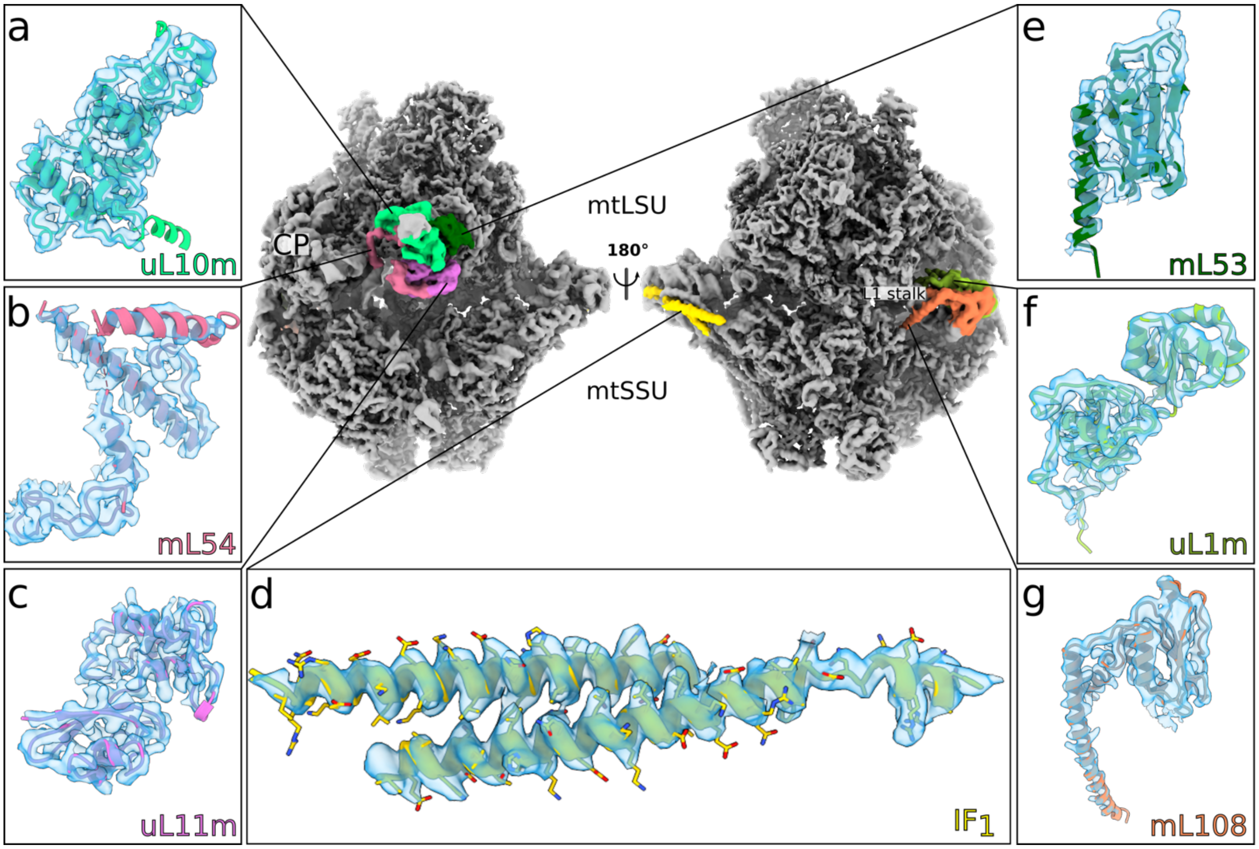

Supplementary Fig. 6. Cryo-EM reconstruction and newly modelled proteins. Two views of a composite cryo-EM map with newly identified proteins colored. The close-up views show the newly modeled proteins with their corresponding density map: **a** uL10m. **b** mL54. **c** uL11m. **d** IF_1. **e** mL53. **f** uL1m. **g** mL108.

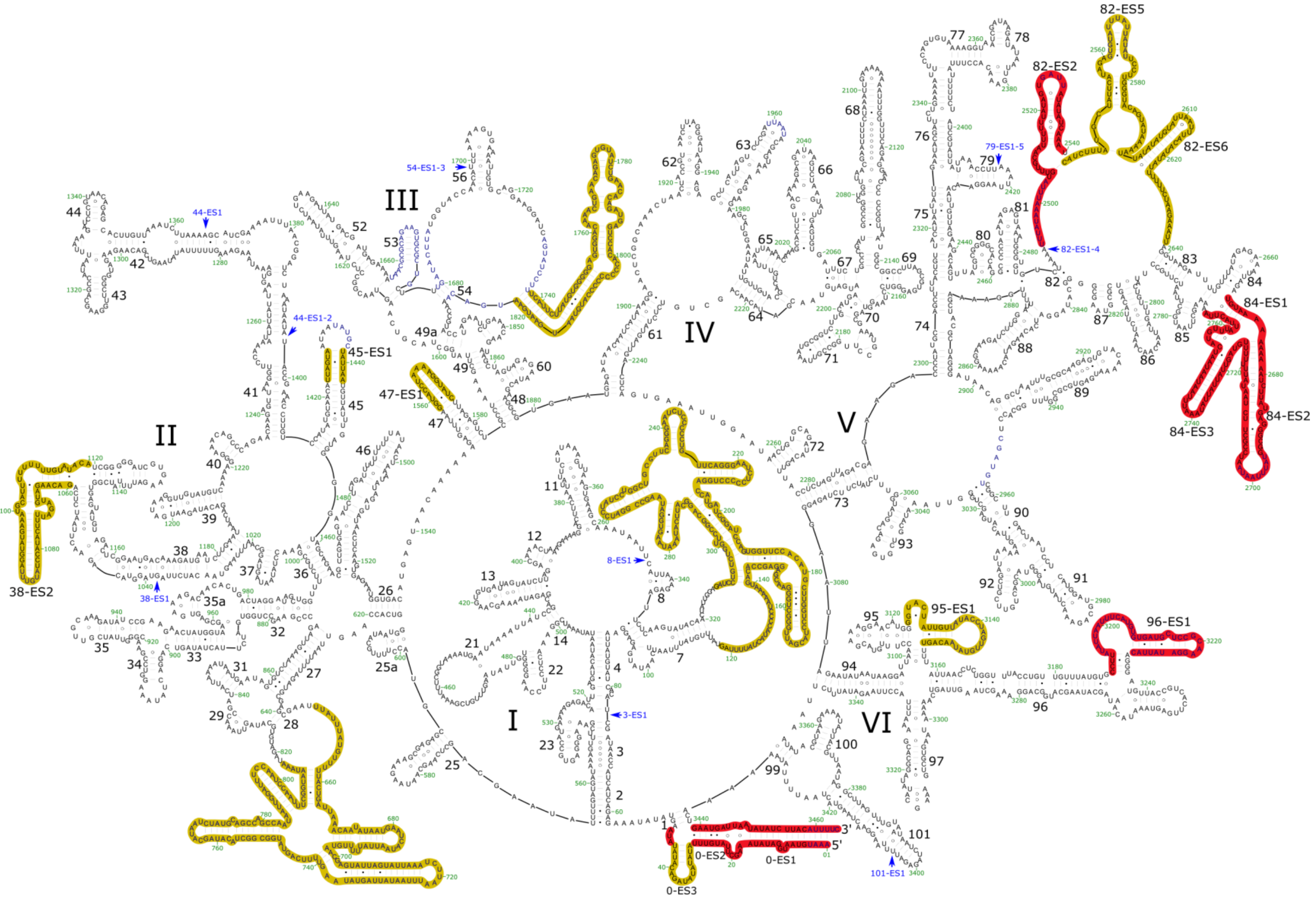

Supplementary Fig. 7: Structure-based secondary structure diagram of the mtLSU rRNA. Expansion segments shared between *N. crassa* and *S. cerevisiae* are highlighted with red, specific expansion segments are highlighted with yellow. Those present in *S. cerevisiae* only are indicated by light blue arrows. rRNA domains are labeled with roman numerals.

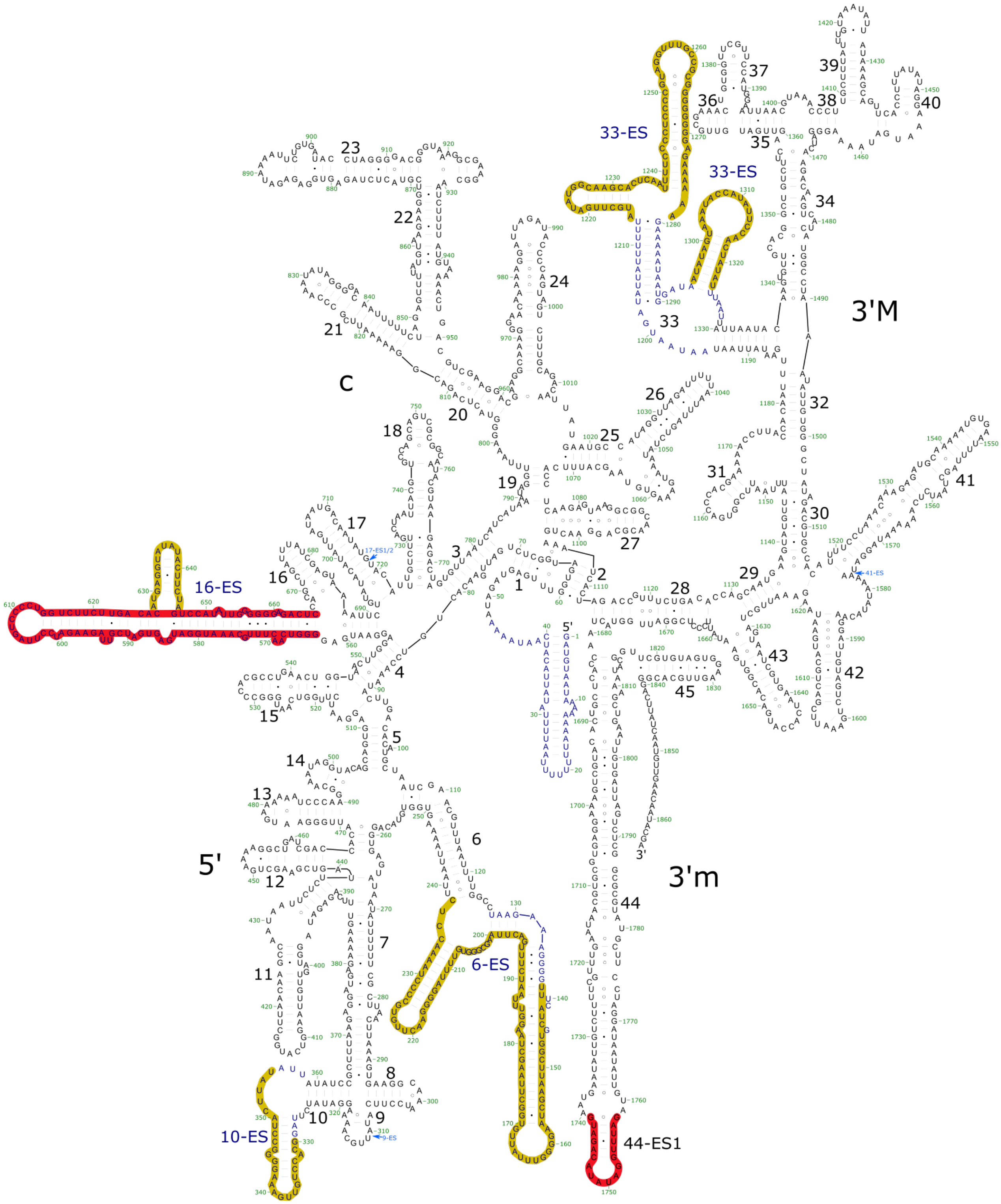

Supplementary Fig. 8: Structure-based secondary structure diagram of the mtLSU rRNA. Expansion segments shared between *N. crassa* and *S. cerevisiae* are highlighted with red, specific expansion segments are highlighted with yellow. Those present in *S. cerevisiae* only are indicated by light blue arrows. rRNA domains are labeled with roman numerals.

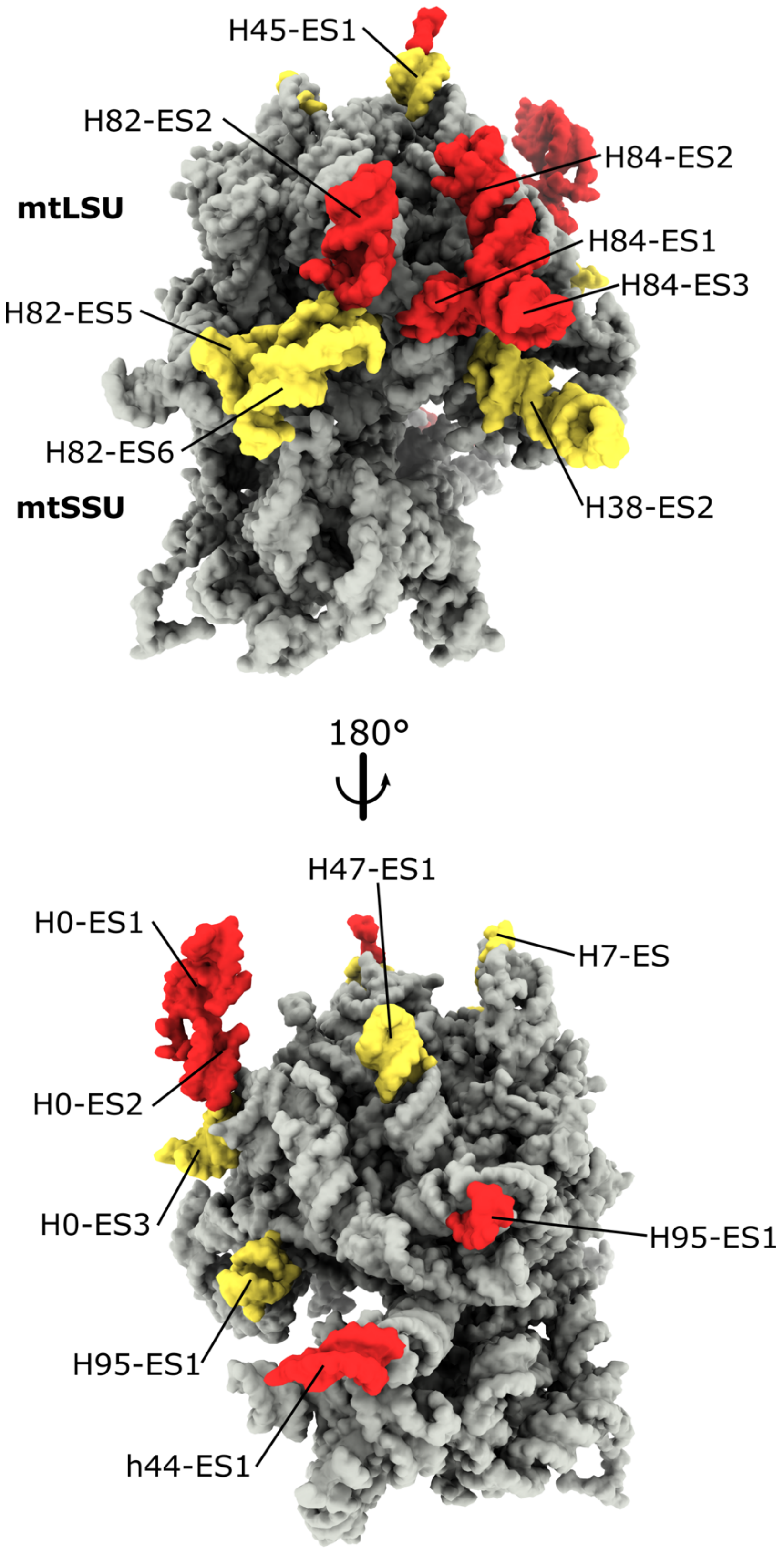

Supplementary Fig. 9: 3D view of the rRNA of mtLSU and mtSSU. The conserved rRNA is in gray. ESs shared with *S. cerevisiae* are in red, and specific ESs in yellow.

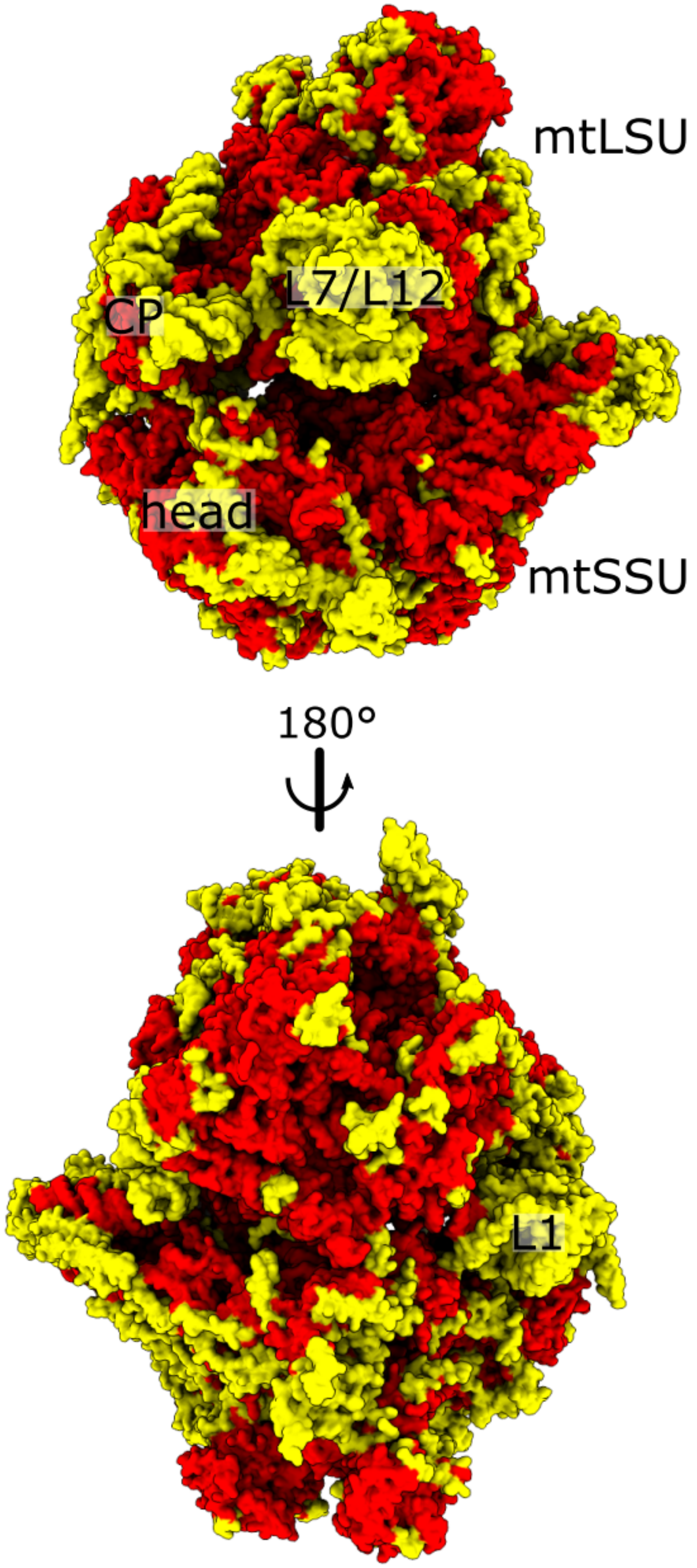

Supplementary Fig. 10: Improvements in the fungal mitoribosomal model. The newly modeled and improved features in the current structure (yellow) compared with the previous model (red) of the *S. cerevisiae* mitoribosome (PDB ID: 5MRC).

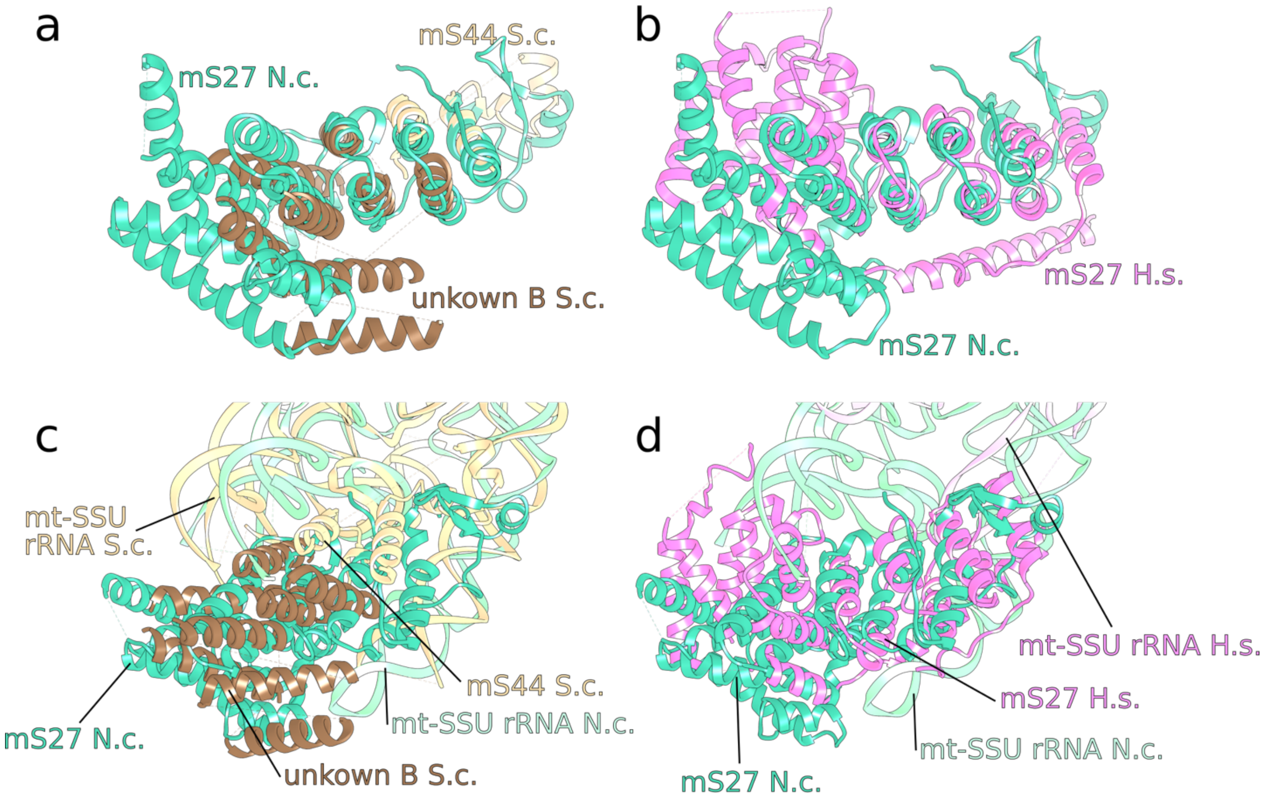

Supplementary Fig. 11: Superposition of mS27 orthologs. a *N. crassa* and *S. cerevisiae* mS27 is superimposed (previously assigned as mS44, 'unknown B'). **b** *N. crassa* and *H. sapiens* mS27 is superimposed, showing similar fold. **c** Superposition based on mtSSU rRNA suggests the same location form mS27 from *N. crassa* and *S. cerevisiae*. **d** Superposition based on mtSSU rRNA suggests the same location form mS27 from *N. crassa* and *H. sapiens.*

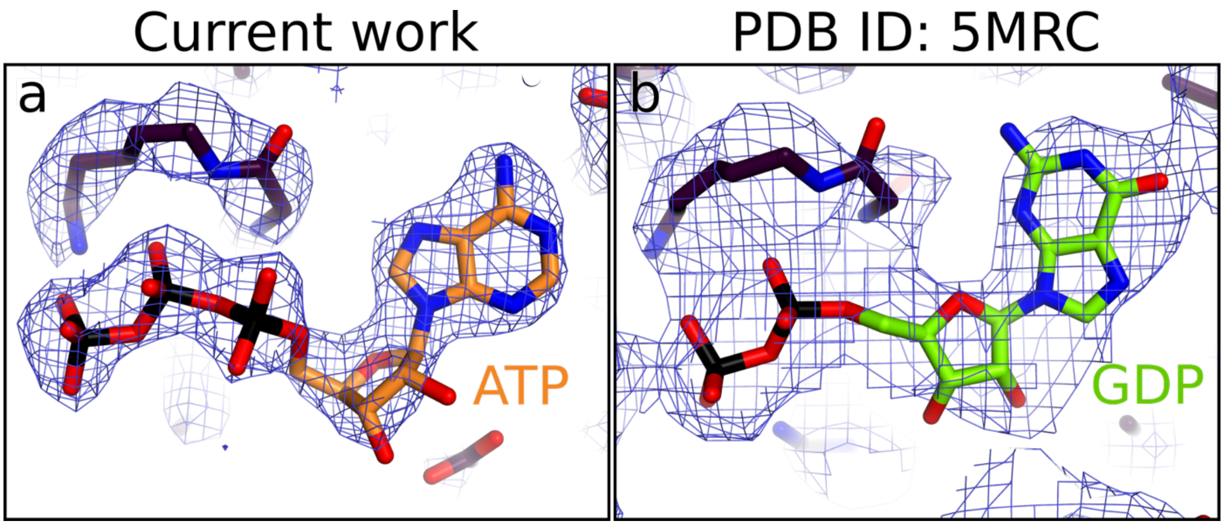

Supplementary Fig. 12: Comparison of the nucleotide density for mS29 between the current model of *N. crassa* and previous work. a The density for ATP bound to mS29 in the current work with the corresponding model **b** For comparison the density of the previously modeled GDP in *S. cerevisiae* is shown.

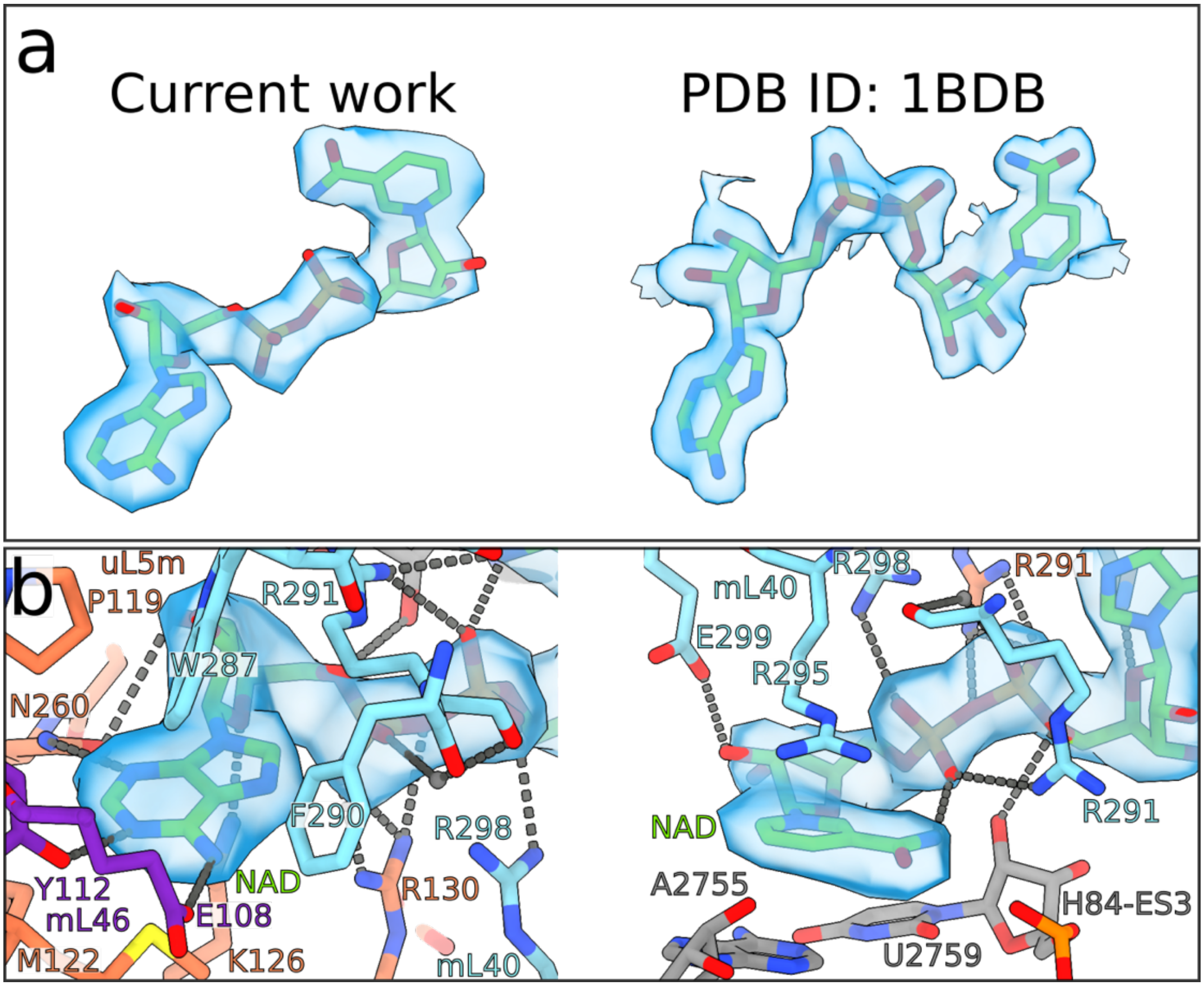

Supplementary Fig. 13: Density map and interactions of the NAD binding pocket in the CP of *N. crassa.* **a** Comparison of the density in the current work with 2 Å resolution crystal structure (PDB ID: 1BDB) confirms the correct assignment of NAD in the model. Both studies show the nicotine amid in syn-conformation. **b** Interactions formed by NAD with uL5, mL40 and H84-ES3 rRNA showed from two opposite views. One view is centered on the adenine ring and the other view around the nicotine amid. Hydrogen bonds and charged interactions are indicated by black dashed lines. Residues and nucleotides are colored and labeled correspondingly.

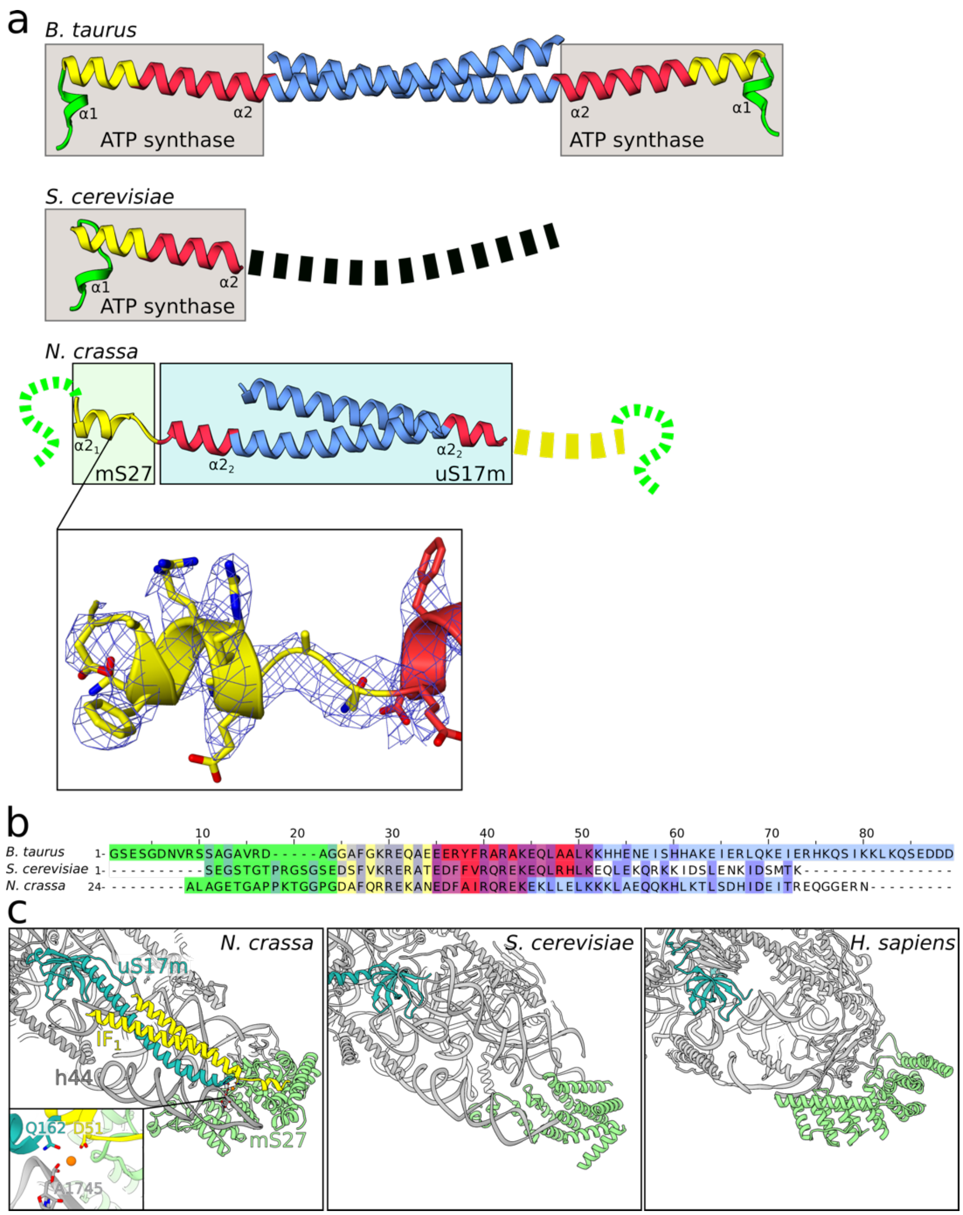

Supplementary Fig. 14: IF$_1$ topology, structure, binding and conservation. a Comparison with *Bos taurus* (generated from PDB IDs: 1GMJ [1] and 4TSF [2]) and *S. cerevisiae* (PDB ID: 3ZIA). Density map with model is shown to illustrate the quality of the data in this region. The mitoribosome and F$_1$-ATPase binding parts are indicated with boxes. For *N. crassa*, the disordered N-terminal region (residues 24-39), the short helix α2$_1$ with a loop (residues 40-49), the longer helix α2$_2$ outside the dimerization part (residues 50-58), and α2$_2$ within the dimerization part (residues 59-88) are green, yellow, red, and blue, respectively. Corresponding residues are colored accordingly in *B. taurus* and *S. cerevisiae*, where α1 is located in the N-terminal region and α2$_1$ and α2$_2$ are a continuous helix α2. **b** Structure-based sequence alignment, colored as in **a**. The mitochondria targeting sequence is included in the residue numbering for *N. crassa*. **c** Structure of the IF$_1$-binding site of *N. crassa* mtSSU and the corresponding regions from *S. cerevisiae* and *H. sapiens*. IF$_1$, uS17m, and mS27 are indicated. The IF$_1$ homodimer forms a helical bundle with the specific C-terminal extension of uS17m. The Mg-ion mediated bridge is shown in a zoom-in window.

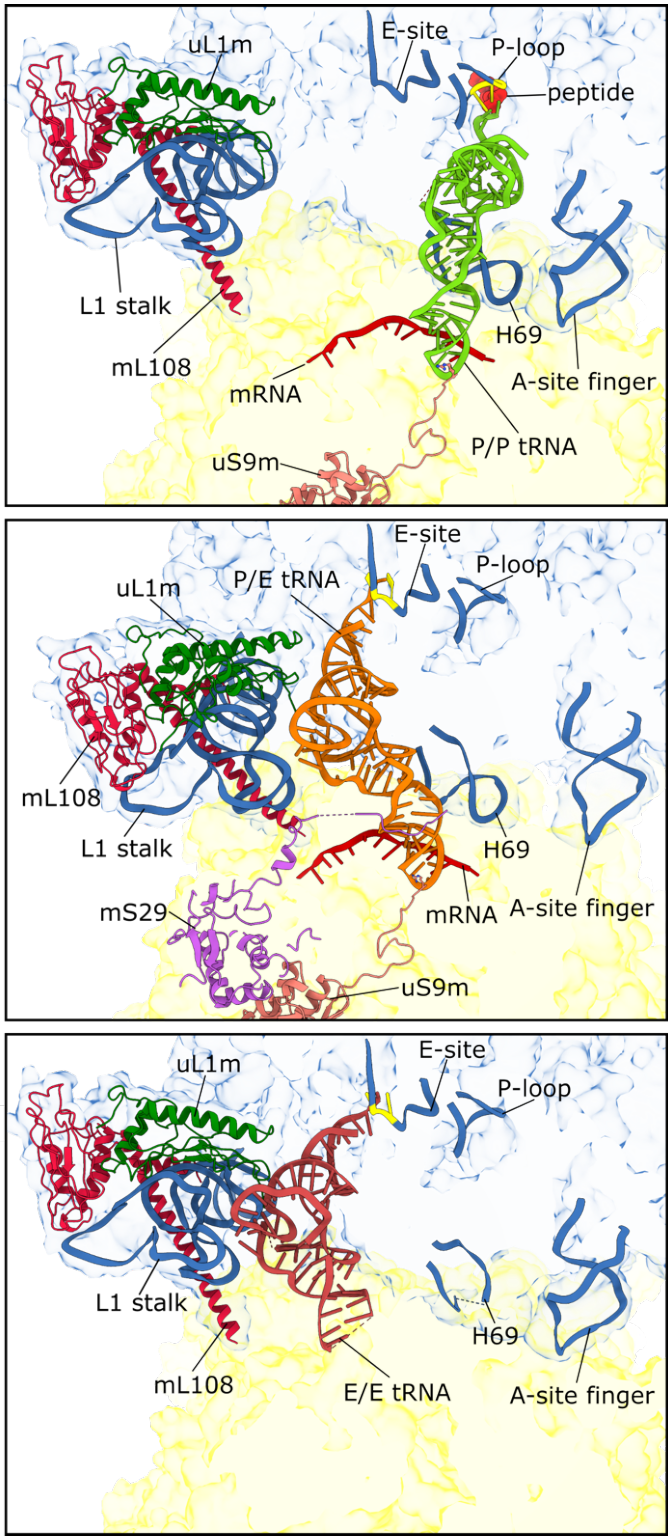

Supplementary Fig. 15: The L1-stalk and translocation of tRNA. The three states are structurally aligned on mtLSU. Transparent surfaces of mtLSU and mtSSU are colored in blue and yellow respectively. Cartoon representations of involved parts are shown and labeled. The P/P-tRNA state with the tRNA colored in green (top). The P/E-tRNA state with the tRNA colored in orange (middle). The E/E-tRNA state with the tRNA colored in red (bottom).

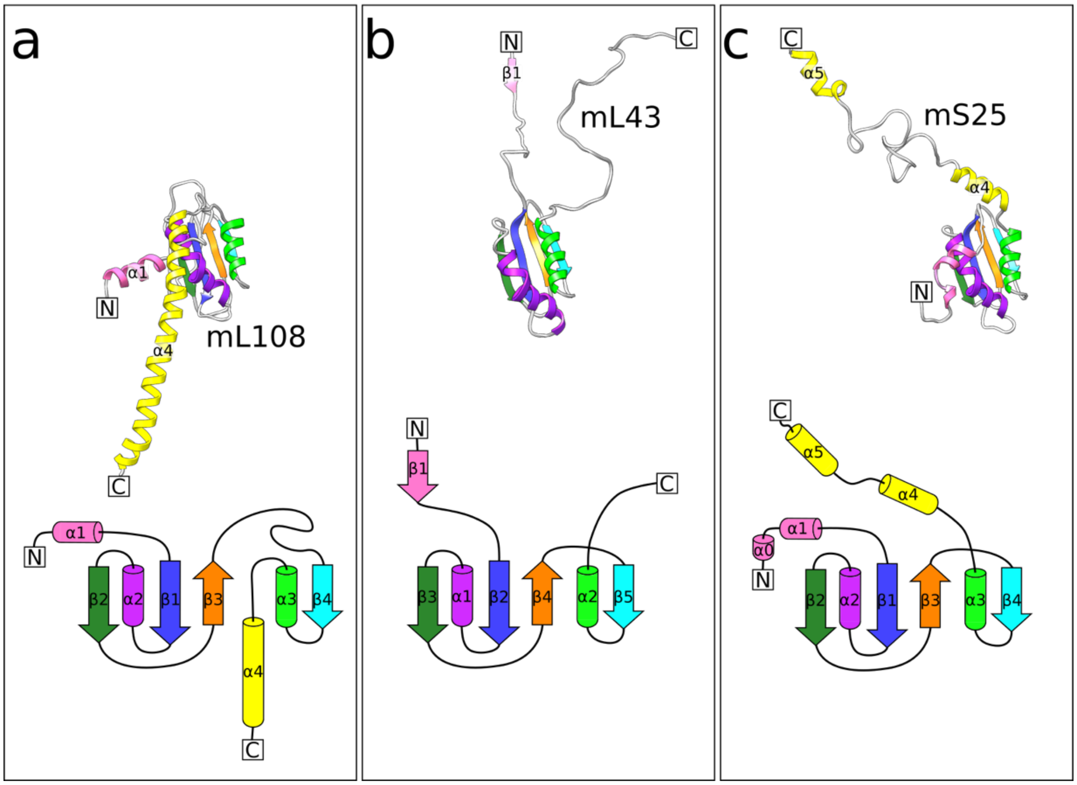

Supplementary Fig 16: Topology of mL108, mL43, and mS25. Three dimensional models and topology of the homologous proteins with secondary structure elements colored, featuring their structural similarity. **a** mL108 of *N. crassa*. **b** mL43 of *N. crassa* and **c** mS25 of *Sus scrofa* (PDB ID: 5AJ4).

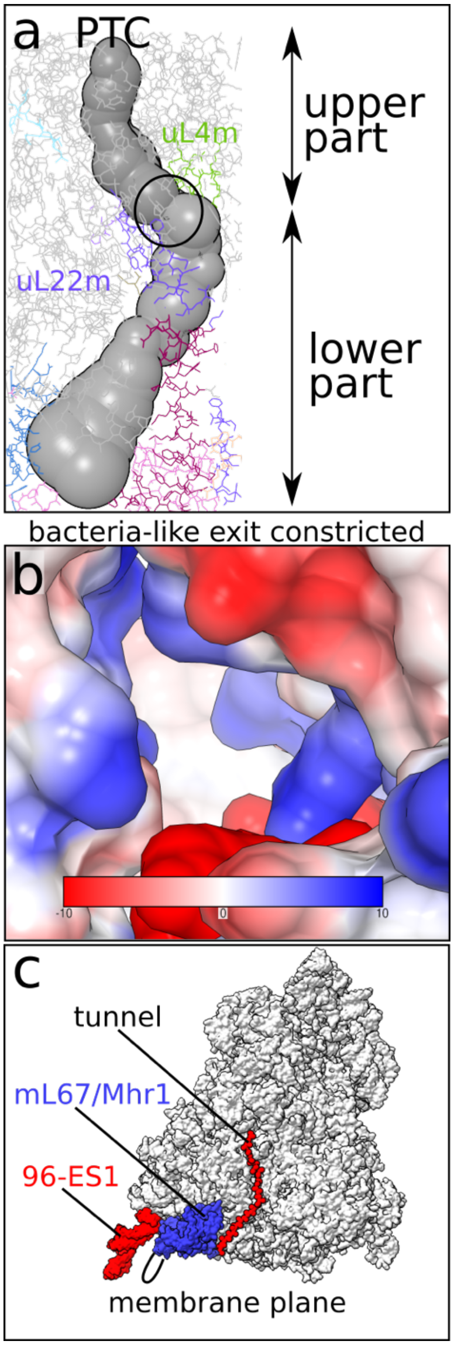

Supplementary Fig. 17: Analysis of potential tunnel exit sites. a The calculated tunnel showing the partition of the tunnel in to the upper and lower parts. The constriction site (circled) is surrounded by uL22m and uL4m. **b** The view of the bacteria-like exit site in surface representation colored by electrostatic Coulomb potential (kcal/mol*e) shows constriction with positively charged protein environment. **c** The polypeptide path in the *N. crassa* mitoribosome. The flexible elements 96-ES1 and mL67/Mhr1 extension around the exit are indicated.

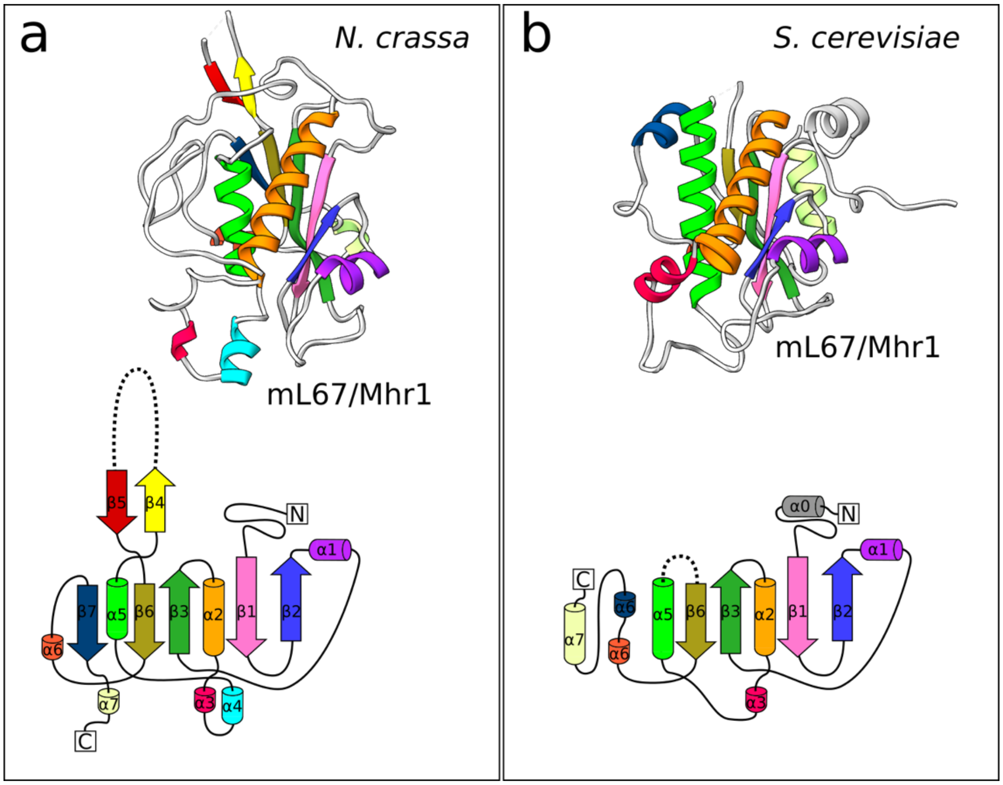

Supplementary Fig. 18: Structure and topology of mL67/Mhr1. a Three dimensional model and topology of *N. crassa* mL67/Mhr1 with secondary structure elements colored. The specific extension between β-strands 4 and 5 is indicated as dashed line. **b** Three dimensional model and topology of *S. cerevisiae* mL67/Mhr1 following the same color scheme in panel **a**.

Supplementary Table 1. Data and model statistics.

**Data collection**	**mtLSU**	**mtSSU**	**Monosome with E-site tRNA**	**Monosome with P-site tRNA**	**Monosome with tRNA in P/E state**	**Late stage intermediate mtLSU (Atp25)**
Microscope	Titan Krios	Titan Krios	Titan Krios	Titan Krios	Titan Krios	Titan Krios
Detector	K2 Summit	K2 Summit	K2 Summit	K2 Summit	K2 Summit	K2 Summit
Magnification	130,000	130,000	130,000	130,000	130,000	130,000
Voltage [kV]	300	300	300	300	300	300
Total electron dose [e^{-}/Å^{2}]	35	35	35	35	35	35
Defocus range [µm]	−0.8 to −3.0	−0.8 to −3.0	−0.8 to −3.0	−0.8 to −3.0	−0.8 to −3.0	−0.8 to −3.0
Pixel size [Å]	1.06	1.06	1.06	1.06	1.06	1.06
Final particles	131,806	131,806	23,802	24,611	37,908	24,142
Resolution [Å] (overall/ LSU core / LSU CP / LSU L10 region / LSU L1 stalk / SSU head / SSU body / SSU tail)	2.74/2.70/2.88/3.03/3.48/-/-/-t	2.85/-/-/-/-/2.79/2.79/2.88	3.10/2.99/3.14/3.50/-/3.14/ 3.07/3.21	3.05/2.97/3.01/ 3.07/-/3.03/ 3.03/3.10	2.99/2.90/3.12/ 3.14/3.48/2.94/ 2.94/3.05	3.03/-/-/-/-/-/-/-
Map-sharpening *B* factor [Å^{2}] (overall/ LSU core / LSU CP / LSU L10 region / LSU L1 stalk / SSU head / SSU body / SSU tail)	-50.7/-48.3/-69.6/-74.4 /-66.7/-/-/-	-51.4/-/-/-/-/-48.3/-56.5/-64.7	-41.2/-33.6/ -60.9/-55.2/-/ -50.4/-32.3/ -55.4	-49.0/-36.8/ -46.0/-56.2/-/ -50.1/-50.1/ -57.0	-45.8/-46.1/ -55.9/-55.9/ -66.7/-41.1/ -45.8/-61.0	-40.3/-/-/-/-/-/-/-
**Model composition**						
Total atoms (all atoms/ hydrogen)	223,408/97,527	167,560/76,668	390,797/173,689	392419/174345	394,205/175,280	207,611/91,129
Chains (RNA/ protein)	1/44	1/35	3/78	4/79	4/80	1/42
RNA residues	2820	1435	4334	4362	4357	2529
Protein residues	8200	7528	15553	15604	15727	7802
Metal ions (Mg^{2+}/ K^{+}/ Zn^{2+})	163/12/2	97/22/0	249/50/2	250/49/2	242/55/2	144/23/2
Ligands (ATP/ NAD/ spermine)	0/1/1	1/0/0	1/1//1	1/1/1	1/1/1	0/1/1
**Refinement**						
Model to map CC (CC_{mask}/ CC_{box}/ CC_{peaks}/ CC_{volume})	0.86/0.76/0.73/0.84	0.86/0.73/0.67/0.85	0.81/0.77/0.75/0.80	0.83/0.78/0.77/0.82	0.84/0.79/0.78/0.83	0.81/0.76/0.74/0.79
Average *B* factor [Å^{2}] (protein/RNA/ metal ion and ligand/)	42.56/39.71/23.92	36.80/36.55/31.32	42.75/53.92/29.43	21.37/21.50/11.39	27.67/29.15/18.25	17.73/25.77/15.02
RMSD bond lengths [Å]	0.002	0.002	0.002	0.002	0.002	0.002
RMSD bond angles [°]	0.382	0.357	0.373	0.354	0.362	0.402
**Validation by MolProbity**						
Clash score	1.57	2.48	1.86	1.75	1.69	1.61
Rotamer outliers [%]	0.23	0.14	0.69	0.41	0.71	0.74

Ramachandran plot [%] (Favored/ allowed/ disallowed)	98.31/1.69/0.00	98.40/1.60/0.00	98.21/1.76/0.03	98.44/1.53/0.03	98.20/1.79/0.01	98.44/1.56/0.00
**EMDB ID** (overall/ LSU core / LSU CP / LSU L10 region / LSU L1 stalk / SSU head / SSU body / SSU tail)	10973/10974/ 10976/10975/-/-/-/-	10958/-/-//-/-/ 10961/10962/ 10963	10978/10979/10981/ 10980/-/10982/ 10984/10983	10985/10986/10989/ 10988/-/10990/ 10991/10992	10965/10966/10968/ 10967/10971/10969/ 10970/10972	10977/-/-/-/-/-/-/-
**PDB ID**	6YWS	6YW5	6YWX	6YWY	6YWE	6YWV

129

Supplementary Table 2. List of RNA and proteins from mtLSU.

Name	Gene	Uniprot ID	Chain ID	Modeled	Size	Notes
23S rRNA	–	–	A	2820	3464	Spermine bound next to A1271.
uL1m	MRPL1	Q1K699	e	59–300	303	Conserved in *S. cerevisiae* and human but disordered in their structures.
uL2m	RML2	Q7SCX7	B	54–379	383	Likewise its *S. cerevisiae* counterpart, NT extension locates at the subunits interface.
uL3m	MRPL9	Q1K8T6	C	63–369	384	A helix at CT extension is similar to the human uL3m. This extension does not exist in the *S. cerevisiae* counterpart.
uL4m	YML6	V5IMN1	D	62–302, 312–324	325	
uL5m	MRPL7	Q1K6P0	E	44–352	352	
uL6m	MRPL6	Q7RZF0	F	52–252	255	
bL9m	MRPL50	Q7S054	G	52–125	300	
uL10m	MRPL11	Q7RZ62	f	54–298	347	Conserved in *S. cerevisiae* and human. It is not modeled in the *S. cerevisiae* structures.
uL11m	MRPL19	Q7RX40	g	12–158	158	Conserved in *S. cerevisiae* and human. It is not modeled in the *S. cerevisiae* structures.
uL13m	MRPL23	Q7SBV6	H	1–183	183	
uL14m	MRPL38	Q7SBJ8	I	1–46, 59–131	131	
uL15m	MRPL10	Q7SB98	J	47–289	312	
uL16m	MRPL16	F5HIJ5	K	61–228	249	
uL17m	MRPL8	Q1K8C8	L	2–193	193	CT extension unlike its *S. cerevisiae* counterpart lacking a helix.
bL19m	IMG1	Q7RYW8	M	41–234	258	
bL21m	MRPL49	Q7SGE5	N	85–217	217	
uL22m	MRPL22	Q7S5N0	O	43–71, 122–364	364	NT extension extensively remodeled with respect to its *S. cerevisiae* counterpart, whereas CT extension is quite similar. NT partially occupies the position of the absent bacterial rRNA helix 24.
uL23m	MRP20	Q7SA60	P	13–192	228	CT extension remodeled respective to its *S. cerevisiae* counterpart.
uL24m	MRPL40	Q7RXU7	Q	1–353	396	NT extension is a long helix that is not like its bacterial, *S. cerevisiae*, and human counterparts. CT extension is similar to that of the *S. cerevisiae*; it covers a long distance through the surface thereby connecting different parts of the large subunit. The CT extension compensates the absence of bacterial helices 15 and 16.
bL27m	MRP7	Q1K730	R	67–196, 248–383	447	NT extension is similar to bacterial, *S. cerevisiae* and human counterparts. CT extension extensively remodeled respective to *S. cerevisiae* bL27m, and partially interacts with rRNA helix 82.
bL28m	MRPL24	Q7SC44	S	42–203, 209–225	274	CT extension remodeled extensively respective to *S. cerevisiae* counterpart.
uL29m	MRPL4	Q7S910	T	44–223	263	
uL30m	MRPL33	Q1K8Y7	U	2–102, 125–161	161	
bL31m	MRPL36	Q1K7L7	V	48–128, 189–207	219	Bridging the mtLSU CP and the mtSSU head.
bL32m	MRPL32	Q1K4P1	W	64–122	129	Zn ion coordination.
bL33m	MRPL39	V5IM60	X	7–54	59	
bL34m	MRPL34	Q96U95	Y	95–140	140	
bL36m	RTC6	Q7S4E7	0	79–124	124	Zn binding

mL38	MRPL35	Q7RXV8	1	83–449	449	CT extension partially compensates the absence of bL35. Stabilizes helices 84-ES and 82-ES.
mL40	MRPL28	V5IQE0	2	248–370	370	Stabilize helices 82-ES, 84-ES, 38-ES1. NAD is binding and interacting with mL40.
mL41	MRPL27	Q7S5W0	3	9–103	103	
mL43	MRPL51	Q7S300	4	2–138	138	
mL44	MRPL3	Q7SA88	5	55–237, 273–439	439	NT extension slightly remodeled respective to *S. cerevisiae* mL44. CT extension interacts with helix 0-ES1. It forms heterodimer with mL57.
mL46	MRPL17	Q7S1Z3	6	82–245 253–303 311–368	368	Interacts with helices 38-ES1 and 82-ES.
mL49	IMG2	Q7S518	7	82–165	165	Most likely interacts with helix 28.
mL50	MRPL13	Q7S711	8	112–442	443	Extensively remodeled. A 31 amino-acid long helix at the solvent-exposed surface of the protein. Interacting with helices 45 and 46.
mL53	MRPL44	Q7SGH0	h	1–98	98	Conserved in *S. cerevisiae* and human. It is not modeled in the *S. cerevisiae* structures.
mL54	MRPL37	Q7SCZ3	i	77–118, 137–218	218	Bridges L10/L11 area to CP. Conserved in *S. cerevisiae* and human. It is not modeled in the *S. cerevisiae* structures and only one helix is modeled in the human structures.
mL57	MRPL15	Q7S1R6	9	61–266	267	Forms a heterodimer with mL44. Stabilizes helix 0-ES1.
mL58	MRPL20	Q1K6U7	a	41–103, 128–225	225	Interacts with helix 0.
mL59	MRPL25	A0A0B0DMU6	b	2–162	162	
mL60	MRPL31	U9W8F2	c	13–110	110	
mL67	MHR1	Q7RYM5	d	33–218 243–291	292	Similar to its *S. cerevisiae* counterpart with slight remodeling at NT and CT extensions.
mL108	MRPL49	Q7RWZ7	j	2-196	201	Conserved but not modeled in *S. cerevisiae*.
Nascent polypeptide	–	–	cc	7		

Supplementary Table 3. List of RNA and proteins from mtSSU.

MRP	Gene	Uniprot ID	Chain ID	Modeled	Size	Notes
16S rRNA	–	–	aa	1435	1864	
bS1m	MRP51	A7UWX2	AA	5–124, 132–213, 229–269, 288–337, 341–358, 368–430	470	
uS2m	MRP4	V5ILE0	BB	107–396	428	
uS3m	VAR1	P23351	CC	17–171, 193–369, 403–508	508	
uS4m	NAM9	Q7SA90	DD	1–215, 379–453	453	
uS5m	MRPS5	Q1K548	EE	56–180, 236–477	477	Has a unique insertion to form a unique second beak.
bS6m	MRP17	Q7SB95	FF	1–117	117	
uS7m	RSM7	Q7S6M9	GG	87–309	309	
uS8m	MRPS8	Q7SHF3	HH	2–161	161	
uS9m	MRPS9	Q7S7R6	II	69–315	315	
uS10m	RSM10	Q7RYL4	JJ	81–268	268	
uS11m	MRPS18	Q7SGU0	KK	253–376	376	
uS12m	MRPS12	Q7S9I4	LL	45–172	174	
uS13m	SWS2	Q7S2C2	MM	2–119	119	
uS14m	MRP2	Q7SF85	NN	2–113	113	
uS15m	MRPS28	Q1K5G1	OO	38–106, 114–320	320	
bS16m	MRPS16	P08580	PP	2–99	107	
uS17m	MRPS17	Q7S4E0	QQ	6–163	165	A unique C–terminal helix, which binds the IF_1 dimer.
bS18m	RSM18	Q1K8E0	RR	108–241	256	
uS19m	RSM19	Q1K8V2	SS	9–89	91	
bS21m	MRP21	Q7SAJ1	TT	149–236	236	
mS23	RSM25	F8MRK5	UU	2–128, 142–238	240	
mS26	PET123	Q7SHR9	VV	47–257, 262–287, 293–316	316	

mS27	MRP13	Q7RYW7	WW	40–270, 275–396	396	Corresponds to mS27 in human. *S. cerevisiae* mS44 and the unknown B (PDB ID: 5MRC) are one connected protein, which corresponds to human and *N. crassa* mS27.
mS29	RSM23 /DAP3	Q7SD06	XX	57–66, 72–469	469	DAP3 death associated protein 3. ATP and Mg ion are bound.
mS33	RSM27	Q1K5R0	YY	2–100	108	
mS35	RSM24	Q1K5Z0	ZZ	42–353	382	Has a unique N–terminal domain to form a unique beak.
mS37	MRP10	Q7S4Y4	11	2–89	90	
mS38	COX24	Q7SHR6	22	312–344	344	
mS41	FYV4	Q1K6Q3	33	43–235	236	
mS42	RSM27 /MRP1	Q7S2H6	44, 55	43–302	310	Homolog of Fe superoxide dismutase. Lost the catalytic Fe ion binding side. Two chains form a homodimer, in contrast to the mS42-mS43 heterodimer in *S. cerevisiae*.
mS45	MRPS35	Q7SHB2	66	66–348	348	
mS46	RSM28	Q7SG49	77	223–401	414	
mS47	MRP5 /EHD3	Q1K7A4	88	42–508	508	3-hydroxyisobutyryl-CoA hydrolase. Probably an active enzyme because its active site is conserved.
ATP–IF$_1$	INH1	V5IRA3	00, 99	39–86	95	ATP synthase inhibitor 1. Forms a homodimer located in the mtSSU tail region.
tRNA	–	–	bb	73		P, E or P/E tRNA state. The density is an average of different tRNA species.
mRNA	–	–	ee	11		mRNA in the P and P/E state. The density is an average of different mRNAs.

Supplementary References

1 Cabezon, E., Runswick, M. J., Leslie, A. G. & Walker, J. E. The structure of bovine IF(1), the regulatory subunit of mitochondrial F-ATPase. *EMBO J* **20**, 6990-6996, doi:10.1093/emboj/20.24.6990 (2001).

2 Bason, J. V., Montgomery, M. G., Leslie, A. G. & Walker, J. E. Pathway of binding of the intrinsically disordered mitochondrial inhibitor protein to F1-ATPase. *Proc Natl Acad Sci U S A* **111**, 11305-11310, doi:10.1073/pnas.1411560111 (2014).
